# Effect of discontinuing antipsychotic medications on the risk of hospitalization in long-term care: a machine learning-based analysis

**DOI:** 10.1186/s12916-025-04304-7

**Published:** 2025-08-20

**Authors:** Mikko Nuutinen, Riikka-Leena Leskelä, Daniela Fialova, Ira Haavisto, Harriet Finne-Soveri, Jokke Häsä, Johanna Edgren, Hein van Hout, Daniel E. da Cunha Leme, John P. Hirdes, Graziano Onder, Rosa Liperoti

**Affiliations:** 1 Nordic Healthcare Group, Helsinki, Finland; 2 Department of Social and Clinical Pharmacy, Faculty of Pharmacy in Hradec Králové, Charles University, Hradec Králové, Czech Republic; 3 Department of Geriatrics and Internal Medicine, 1st Faculty of Medicine, Charles University, Prague, Czech Republic; 4 Finnish Institute for Health and Welfare, Helsinki, Finland; 5 VUMC – University Medical Center Amsterdam, Amsterdam, The Netherlands; 6 School of Public Health Sciences, University of Waterloo, Waterloo, ON Canada; 7 Università Cattolica del Sacro Cuore, Rome, Italy; 8 Fondazione Policlinico Universitario A. Gemelli IRCCS, Rome, Italy

**Keywords:** Antipsychotic medications, Long-term care, Machine learning

## Abstract

**Background:**

Antipsychotic medications are frequently prescribed to older residents of long-term care facilities (LTCFs) despite their limited efficacy and considerable safety risks. While discontinuation of these drugs might help reduce their associated morbidity, the impact of stopping antipsychotics on the risk of hospitalization has not been studied yet. The study aimed at estimating the effect of antipsychotic discontinuation on the risk of hospitalization in older LTCF residents and at identifying relevant factors influencing such effect.

**Methods:**

For this registry-based retrospective cohort study, data from a cohort of older LTCF residents in Finland from the years 2014 to 2018 was analyzed. Data sources were the Resident Assessment Instrument for Long-Term Care (RAI-LTC) based comprehensive geriatric assessments and the Finnish Care Register for Health Care. For the initial cohort, 5467 users of antipsychotic medications with at least four assessments, each conducted 6 months apart, were selected. Residents were defined either as discontinuing, if antipsychotics were prescribed at the first two assessments but not at the last two, or as chronic users, if antipsychotics were prescribed at all four assessments. Causal machine learning (ML) methods including double machine learning (DML), double robust (DR), X-learner, and causal forest (CF) were applied to estimate the effect of antipsychotic discontinuation on the risk of hospitalization and to identify factors influencing such effect. The follow-up time was 1 year. The methods of SHAP values (SHapley Additive exPlanations), partial dependence plots (PDP), and surrogate models were used for model interpretation.

**Results:**

Nearly 43% of residents in the study discontinued antipsychotic medications. Antipsychotic discontinuation lowered the probability of hospitalization of about 12% (average treatment effect, ATE). The individual treatment effect (ITE) estimations ranged from − 30% to + 1%. The use of restraints, age, and functional impairment were relevant variables in all ITE models in influencing the predicted ITE.

**Conclusions:**

Antipsychotic discontinuation may decrease the likelihood of hospitalization among older LTCF residents, benefiting most users of these drugs. Promoting antipsychotic discontinuation may prevent hospitalizations and reduce morbidity and mortality in long-term care.

**Supplementary Information:**

The online version contains supplementary material available at 10.1186/s12916-025-04304-7.

## Background

The use of antipsychotic medications is frequent in long-term care. From 12% to over 50% of older residents of long-term care facilities (LTCFs) in western countries receive an antipsychotic agent with large variation in prescription rates according to geographical areas and over time [1, 2]. In most cases, such prescriptions are outside the approved indications. In fact, with the only exception of risperidone, a second-generation agent that has been also approved for the treatment of dementia-related agitation and aggression in Europe and in Canada, the other antipsychotic medications are approved for the treatment of schizophrenia and other psychotic disorders [3]. However, they are largely used off-label for controlling behavioral and psychological symptoms of dementia (BPSD) such as agitation, aggression, wandering, inappropriate behavior, and sleep disturbances. Prescribing antipsychotic medications for the treatment of BPSD is common practice in spite of evidence of limited efficacy from clinical trials in older adults with dementia and serious safety concerns related to their use [4]. Antipsychotic medications may cause relevant side effects even at therapeutic doses. Their use is particularly cumbersome in older adults with multimorbidity and polypharmacy who have increased susceptibility to develop related adverse effects such as extrapyramidal syndrome, falls, sedation, delirium, and anticholinergic effects [5]. Over the past decade, drug regulators in several countries have issued warnings regarding an increased risk of acute cerebrovascular events and death in older adults taking antipsychotic medications [6]. In response to these concerns, current clinical recommendations advise clinicians to avoid the use of antipsychotic medications in older adults with dementia or limit prescribing them to patients with severe BPSD after a careful evaluation of the related risks and benefits, and for a short period of time [7].

The relationship between antipsychotic discontinuation and the risk of hospitalization among older adults, particularly those residing in LTCFs, is complex. On one hand, discontinuing antipsychotics may reduce the likelihood of acute hospital admission by reducing the risk of drug adverse events such as stroke, falls, or cardiac complications. On the other hand, antipsychotic withdrawal may lead to BPSD exacerbation, increased caregiver burden, or clinical instability, which could also trigger hospital admissions.

Antipsychotic discontinuation may also reduce mortality, potentially increasing the likelihood of hospitalization over time due to longer survival and multiple comorbidities. However, death may itself occur during or as a result of a hospitalization. Recently, clinical trials have shown that deprescribing antipsychotic medications among LTCF residents with dementia has almost no effect on the severity and frequency of symptoms, thus suggesting the appropriateness of regular discontinuation trials in clinical practice [8–10]. To our knowledge, no studies so far have investigated the effect of antipsychotic discontinuation on the risk of hospitalization among LTCF residents.

In this study, we applied causal machine learning (ML) methods including double machine learning (DML) [11], double robust (DR) [12], X-learner [13], and causal forest (CF) [14] to estimate the effect of antipsychotic discontinuation on the risk of hospitalization in a population of older LTCF residents and to identify relevant factors influencing such effect. ML-based methods were chosen to account for the high clinical heterogeneity among older adults in LTCFs. Indeed, traditional statistical approaches—which typically estimate average treatment effects across entire cohorts—may fail to capture the substantial individual variation in this population. To address this, we adopted a causal ML approach based on individualized treatment effect (ITE) models. These models are specifically designed to estimate treatment heterogeneity at the individual level and are particularly well suited for high-dimensional healthcare data. By leveraging these methods, it is possible to move beyond population-level associations and instead provide patient-specific estimates, which may better inform clinical decision-making in real-world settings.

## Methods

### Study design and data

The current report adheres to the Strengthening the Reporting of Observational Studies in Epidemiology (STROBE) statement for cohort studies [15].

This study is a registry-based retrospective cohort study that was conducted as part of the analytical tasks of the I-CARE4OLD project, a European Union (EU) funded program aimed at improving prognostication in older adults with complex chronic conditions through the use of ML methods [16]. The study was approved by the Finnish Institute for Health and Welfare (THL) (permission no. THL/1118/6.02.00/2021). The data sources were the RAI-LTC (Resident Assessment Instrument for Long-Term Care) based comprehensive geriatric assessments of LTCF residents and the Finnish Care Register for Health Care. Trained assessors, usually registered nurses, collected data using the Minimum Data Set (MDS) 2.0 version of the RAI-LTC instrument. All LTCF residents in Finland are regularly assessed with this instrument at least twice per year, as defined by the Elderly Care Act 980/2012. RAI assessments are delivered twice a year to the THL either by the service provider themselves or by their authorized application provider. The national RAI database is maintained by THL, responsible by legislation for keeping social and health records based on the Act on the Institute of Health and Welfare 31.10.2008/668. The validity and reliability of the RAI-LTC instrument has been demonstrated in previous studies [17].

Data collected over the years 2014 to 2018 were used in the present study. The RAI-LTC instrument collects information on each resident’s demographic, functional, medical, and cognitive status and drug prescriptions. Several scales to measure clinically relevant indicators such as cognition (cognitive performance scale, CPS) [18], self-function (activity of daily living, ADL) [19], behavior (aggressive behavior scale, ABS) [20], or depression (depression rating scale, DRS) [21] are embedded in the instrument.

Data preprocessing steps and the operational definitions of variables and scales are described in details in the supplementary material (see Additional file 1) [22–26].

### Definition of study groups

Antipsychotic use was identified from the RAI-LTC section dedicated to drug prescription using the ATC code N05A, excluding N05AN. According to previous estimates from RAI data, the overall prevalence of antipsychotic use among LTCF residents in Finland ranges from 28% to 35% with atypical antipsychotics being the most frequently prescribed agents and risperidone accounting for the majority of prescriptions followed by quetiapine and olanzapine [27]. Residents who were 65 years of age or older were selected for this study. To be included in the study, residents had to have at least four consecutive RAI assessments, each conducted at 6-month intervals. Time period of assessments 1 and 2 was defined as the baseline period. Follow-up period started from the third assessment. Residents were classified in the discontinuing group, if antipsychotic medications were prescribed at the baseline period (assessments 1 and 2) but not at the follow-up period (assessments 3 and 4). Residents were classified in the group of chronic users, if antipsychotic medications were prescribed both at the baseline and follow-up period (assessments 1 to 4). The input variables (or candidate predictors) in the models were collected from the second assessment of the baseline period. For residents with more than one valid group of four RAI assessments, the assessment group was randomly selected. Those residents who died during the study period were excluded from the analyses. Definition of study groups is further described and illustrated in Additional file 1: Fig. S1.

### Definition of study outcome

The main outcome in this study was hospitalization for any cause. Information on hospitalizations was obtained from the Finnish Care Register for Health Care. The operational definition adopted was any number of hospitalizations within 360 days of the first follow-up assessment (i.e., the third assessment) and it was categorized on a binary scale (yes/no).

### Individual treatment effect models

**Fig. 1:**
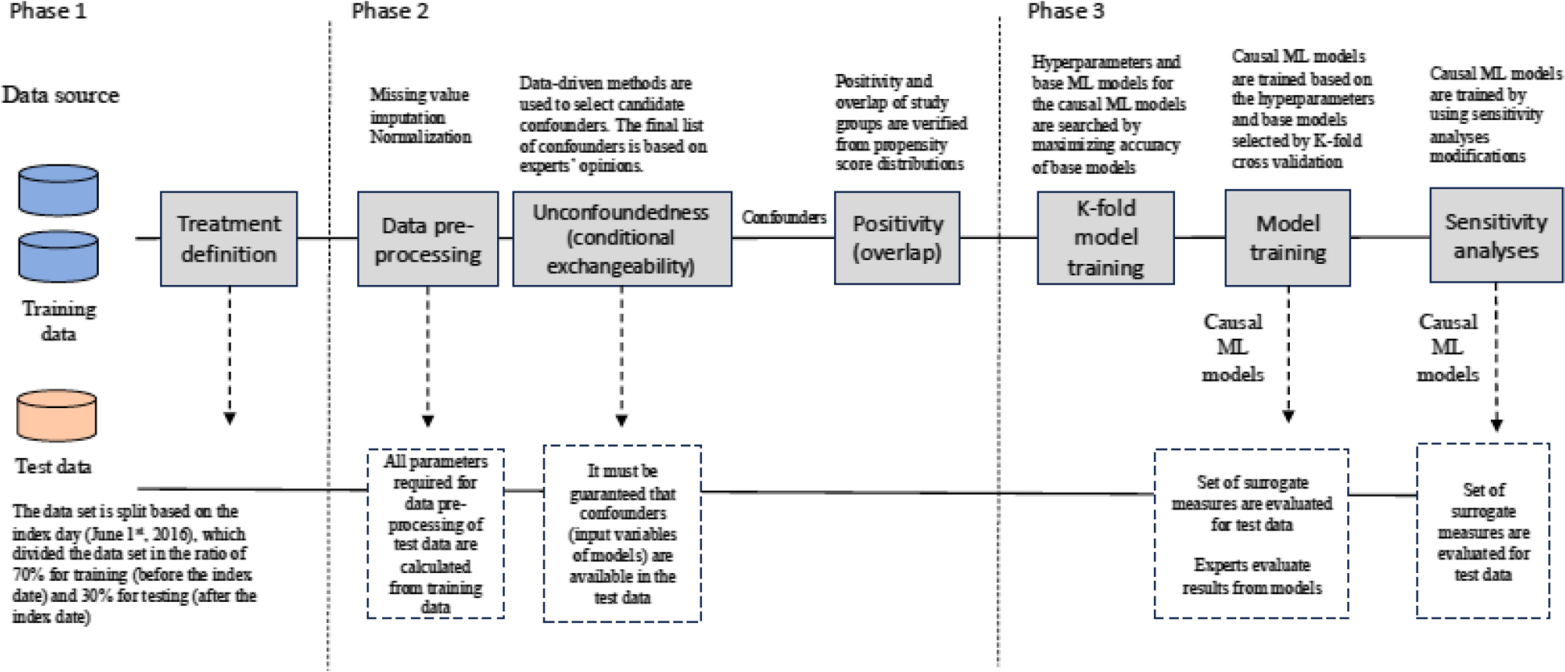
Workflow of ML model training and evaluation

A causal ML approach was adopted to assess the effect of antipsychotic discontinuation on the risk of hospitalization. Causal ML models, particularly ITE models, estimate how an intervention would affect outcomes at the individual level. Unlike standard supervised ML models, which predict the risk of an outcome, ITE models aim to estimate the causal effect of a treatment. This makes them well-suited for evaluating pharmacological and non-pharmacological interventions in older adults with complex chronic conditions, as they account for patient heterogeneity and enable personalized effect estimates. The ITE when antipsychotic medication is stopped can be represented by equation:

1 $$\tau (x)=E\left[{Y}_{i}\left(1\right)-{Y}_{i}\left(0\right)|{X}_{i}=x\right]$$

where *Y*_*i*_(1) and *Y*_*i*_(0) are potential outcomes [28] after the medication is discontinued or continued and *X*_*i*_ are the covariates of resident *i*. This measure (ITE) can be interpreted as the absolute risk reduction (ARR). For example, it can be interpreted that $\widehat{\tau }<0$ indicates that discontinuing antipsychotic medications reduces the risk of hospitalization, while $\widehat{\tau }>0$ implies an increased the risk. Currently, there is no generally accepted standard algorithm for estimating ITE. Therefore, we used several different algorithms (DML, DR-learner, X-learner, and causal forest) and compared their estimates. For training and evaluating causal ML models, the dataset was split into the training/validation set and test set. The split was based on the index day (June 1, 2016), which divided the data set in the ratio of 70% for training/validation (before the index date) and 30% for testing (after the index date). The parameters of the models were searched and the models were trained in the training/validation set. Then, the trained models were evaluated on the test set. The workflow of causal ML model training and evaluation and confounder selection is illustrated in Fig. 1.

### Confounders

From an overall set of 298 variables available from the data source, a subset of potential confounders was selected using both a data-driven and knowledge-based selection approach. A complete list of processed variables has been included in Additional file 1: Table S1. Based on data-driven approach, candidate confounders were searched through univariate logistic regression models that were trained for predicting hospitalization and exposure. The relevance sorting criterion was the area under curve (AUROC) value for both outcomes. A group of three study researchers (DF, HF, RL), who are experts in the field of clinical geriatrics and clinical pharmacy, reviewed the list of potential confounders and included additional variables that, although not considered relevant based on the logistic regression, were considered potential confounders because they were deemed good proxies for unmeasured factors associated with antipsychotic discontinuation and influencing the probability of being hospitalized. The final list of potential confounders included: age, gender, body mass index (BMI), number of medications, number of comorbidities, cognitive decline (CPS score) [18], functional status (ADLH score) [19], depression (DRS score) [21], presence and severity of behavioral symptoms (ABS score) [20], delirium symptoms, delusions or hallucinations, unsteady gait, acute episode or flare-up of recurrent or chronic problem or monitoring acute medical condition, recent hospital visits or emergency department visits, chemotherapy or end-stage disease, problems with eating and swallowing, any restraints used, physician visits in last 14 days or doctor orders changed or abnormal laboratory tests. Detailed definitions of confounders can be found in Additional file 1: Table S2.

### Model evaluation

A fundamental problem in evaluating causal inference models is that a given individual can never be observed in both treated and untreated conditions (Eq. 1). Therefore, metrics that calculate the difference to true treatment effect can only work in a simulation where you know both possible outcomes. However, we can accept that if model found heterogeneity in the data, then model-assisted recommendations are better than random treatment assignment. In this study, we used the area under uplift curve (AUUC) [29] and c-for-benefit [30] metrics to verify this property. Both metrics have been increasingly adopted in the literature to evaluate the discriminative ability of ITE models [31]. Furthermore, we analyzed treatment effect distributions for presenting information about what models have learned from data and conducted a set of sensitivity analyses.

### Model interpretation

For the interpretation ITE models, we used SHAP values (SHapley Additive exPlanations) [32, 33], partial dependence plots (PDP) [34], and surrogate models [35]. PDP plots were calculated for the variables with the highest absolute sum of SHAP values. Our surrogate models were decision trees that were trained to predict the estimations of the trained ITE models when the input were the confounders.

### Software

All analyses were performed using Python version 3.9.7 and the following libraries: Scikit-learn package [36] version 1.0.2 for all data processing steps, EconML [37] version 0.14.0 for ITE models, and SHAP [32, 33] version 0.40.0 for model interpretation.

## Results

From the overall population (*n* = 53,513), a total of 5467 residents on antipsychotic medications had at least four assessments and fulfilled inclusion criteria. Of these, 2348 (42.9%) residents discontinued antipsychotic medications and 3119 (57.0%) residents continued antipsychotic treatment at follow-up. Over two thirds of residents in the sample were women and mean age was 83 years. On average, both antipsychotic discontinuing residents and chronic users had nearly four comorbidities and were on over nine different medications per day. As reported in Table 1, the two study groups were comparable with respect to socio-demographic, clinical, cognitive, and functional characteristics.

**Table 1:** Socio-demographic, functional, cognitive, and clinical characteristics of residents who discontinued antipsychotic medications and chronic users at the baseline period (second assessment)

Item name	All	Chronic user group	Discontinuing group
Residents, no	5467	3119	2348
Age, mean (SD), years	83.3 (7.5)	83.6 (7.6)	82.9 (7.3)
Female gender, no. (%)	3799 (69.5)	2087 (66.9)	1716 (73.1)
BMI, mean (SD), kg/m^2^	25.3 (5.3)	25.3 (5.4)	25.3 (5.3)
Number of drugs, mean, (SD)	9.5 (3.5)	9.8 (3.6)	9.2 (3.4)
Number of comorbidities, mean, (SD)	3.6 (1.8)	3.7 (1.9)	3.4 (1.8)
CPS score, mean, (SD)	3.6 (1.5)	3.5 (1.5)	3.8 (1.5)
ADLH score, mean, (SD)	3.7 (1.7)	3.5 (1.7)	3.8 (1.6)
DRS score, mean (SD)	2.7 (2.7)	2.9 (2.9)	2.4 (2.5)
Severe or very severe behavior problems (ABS > 2), no. (%)	2154 (39.4)	1304 (41.8)	852 (36.3)
Delirium symptoms, no. (%)	4762 (87.1)	2723 (87.3)	2038 (86.8)
Delusions or hallucinations, no. (%)	1799 (32.9)	1163 (37.3)	636 (27.1)
Unsteady gait, no. (%)	2050 (37.5)	1201 (38.5)	850 (36.2)
Flare-up or monitoring of acute condition, no. (%)	142 (2.6)	90 (2.9)	52 (2.2)
Hospital or ER visits (last 6 months), no. (%)	1038 (19)	602 (19.3)	437 (18.6)
Chemotherapy or end-stage disease, no. (%)	11 (0.2)	9 (0.3)	5 (0.2)
No problems with eating and swallowing, no. (%)	4117 (75.3)	2317 (74.3)	1801 (76.7)
Any restraints used, no. (%)	3532 (64.6)	1921 (61.6)	1608 (68.5)
Physician visits, doctor orders changed or abnormal lab values, no. (%)	2323 (42.5)	1310 (42.0)	1014 (43.2)

*Abbreviations* *SD* standard deviation, *BMI* body mass index, *CPS* cognitive performance scale, *ADLH* activities of daily living hierarchy, *DRS* depression rating scale, *ABS* aggressive behavior scale

### Individual treatment effect models

**Fig. 2:**
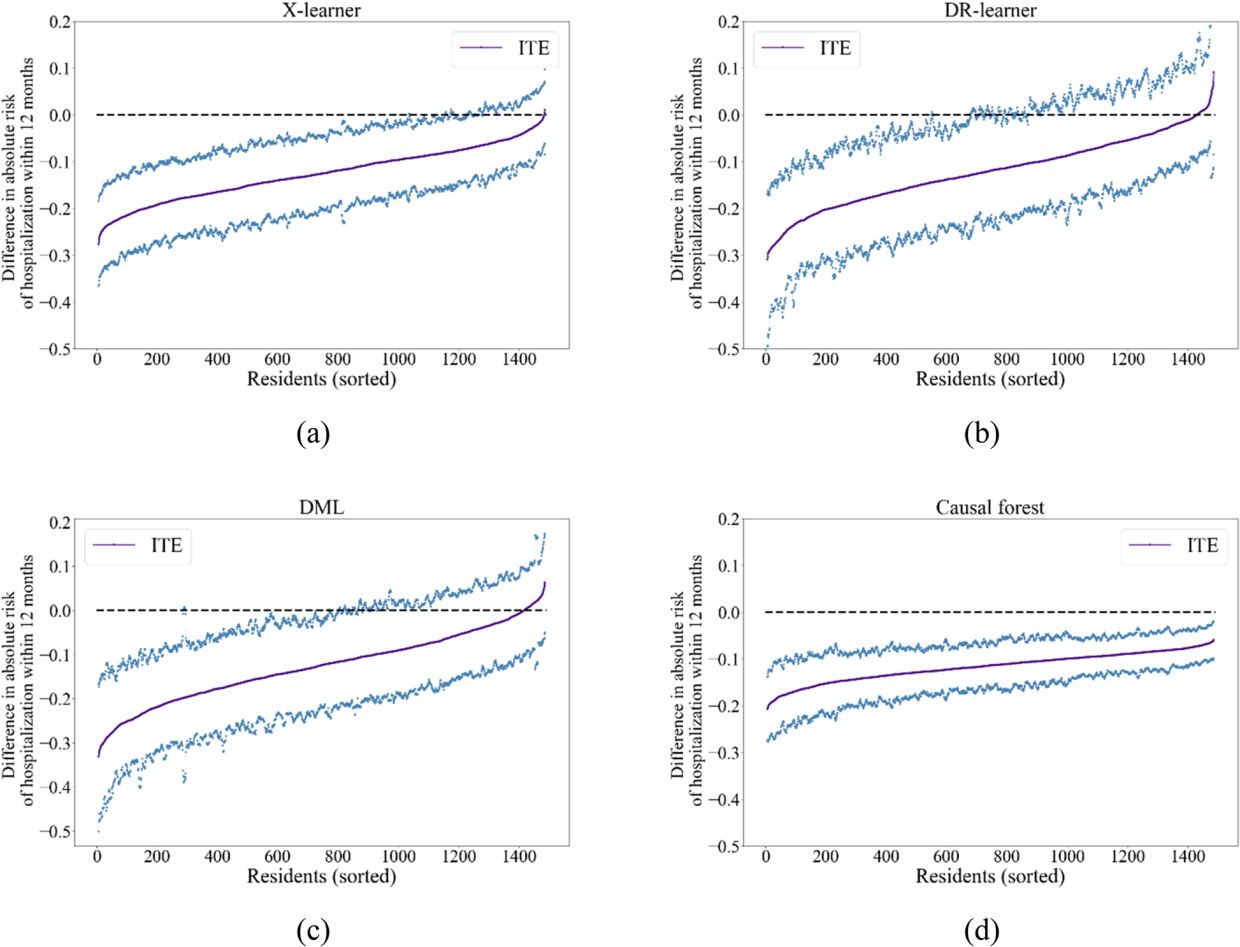
The 95% confidence intervals for the estimates of individual-level treatment effects, ordered by the magnitude of the estimates of **a** X-learner, **b** double robust (DR) learner, **c** double machine learning (DML), and **d** causal forest model

The distributions of the estimated ITEs from ITE models for test data were skewed to negative values. The scale of ITE estimations was from − 30% to + 1% (as illustrated in Additional file 1: Fig. S2). Average treatment effect (ATE) values of different ITE models are presented in Table 2. According to these results, discontinuation of antipsychotics lowered the probability of hospitalization by about 12% (ATE from different models ranging from 11.7% to 12.7%). Figure 2 presents the 95% confidence intervals for the estimates of individual-level treatment effects from ITE models, ordered by the magnitude of the estimates. ITE estimates for hospitalization ranged from − 33% to + 9%. These estimated ITEs suggest that discontinuation of antipsychotics reduces the risk of hospitalization in 95 to 99% (depending on the ITE model) of residents in our test set. Among these residents, 49.3 to 99.4% (depending on the ITE model) had negative ITE estimates with CIs below zero, suggesting that, with a good degree of confidence, discontinuation of antipsychotic medication would reduce the risk of hospitalization based on the DML, DR-learner, X-learner, or causal forest models.

**Table 2:** Average treatment effect (ATE), area under uplift curve (AUUC), and c-for-benefit values for double machine learning (DML), double robust (DR) learner, X-learner, and causal forest models

Algorithm	ATE [CI95%]	AUUC [CI95%]	C-for-benefit [CI95%]
DML	− 0.127 [− 0.232, − 0.015]	0.054 [0.052, 0.057]	0.537 [0.530, 0.545]
DR-learner	− 0.120 [− 0.229, 0.000]	0.040 [0.038, 0.043]	0.523 [0.514, 0.531]
X-learner	− 0.126 [− 0.206, − 0.046]	0.038 [0.035, 0.040]	0.539 [0.531, 0.546]
Causal forest	− 0.117 [− 0.168, − 0.066]	0.063 [0.060, 0.065]	0.548 [0.541, 0.555]

**Fig. 3:**
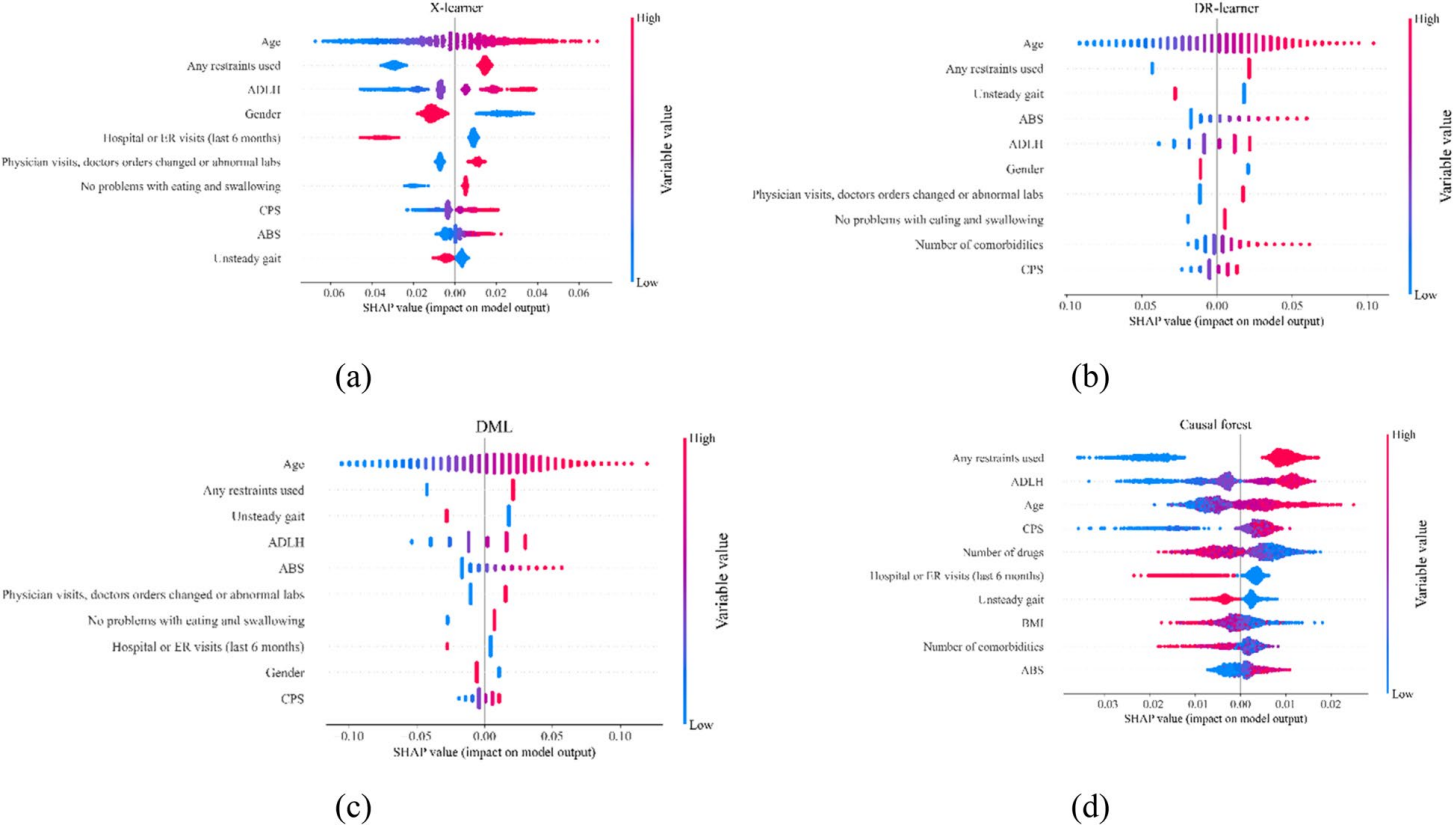
Plots of the impact of baseline variables (SHAP values) on the estimated individual treatment effect (ITE) of **a** X-learner, **b** double robust (DR) learner, **c** double machine learning (DML), and **d** causal forest model

**Fig. 4:**
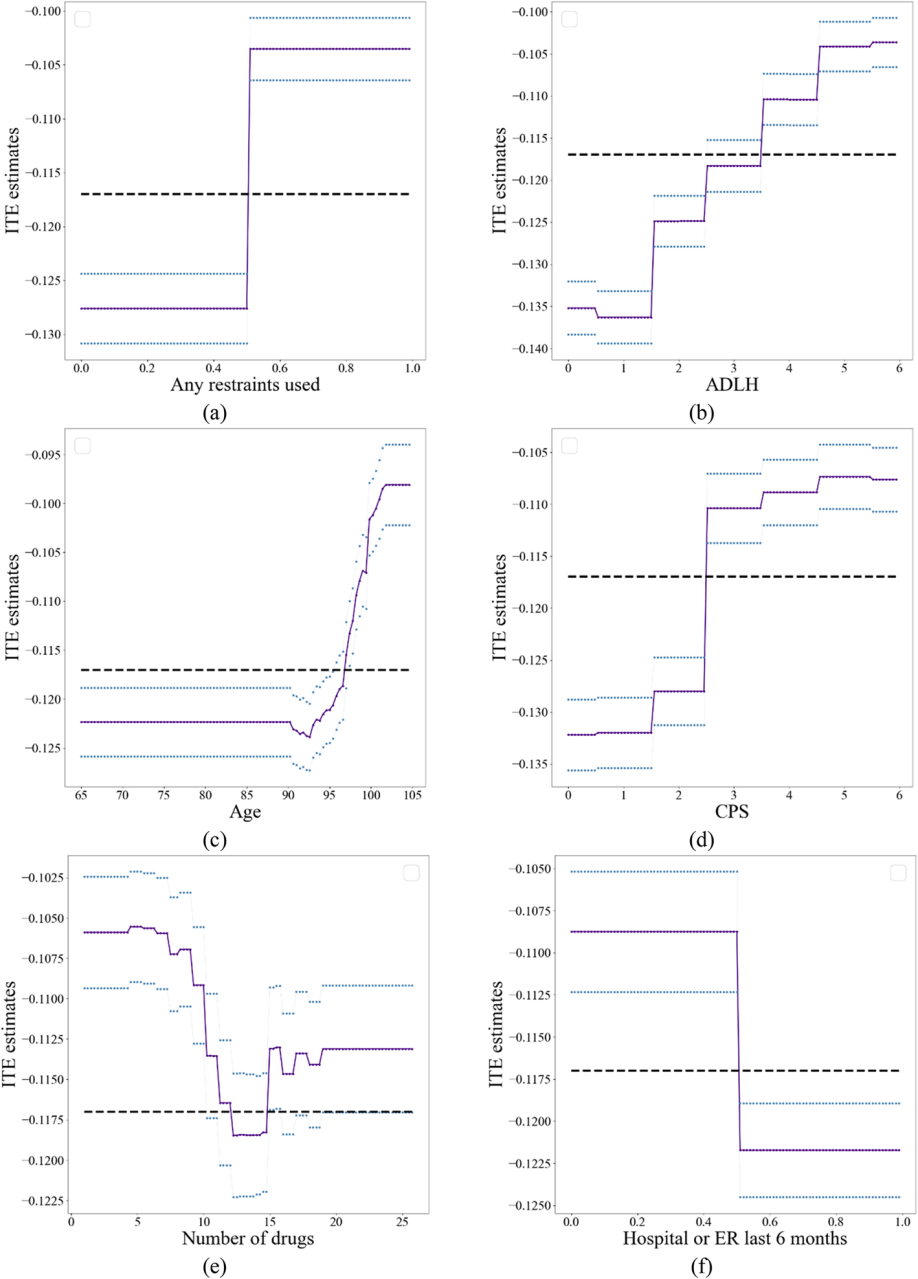
Partial dependence plots (PDP) of the impact of the most relevant variables influencing individual treatment effect (ITE) values from the causal forest model

The distributions of the data points (SHAP values) in the plots in Fig. 3 show the impact of baseline variables on the estimated ITE. The red points indicate higher variable values than the average value of that variable, and the blue points lower variable values than the average value of that variable. Longer distance between red and blue points indicates higher effect of a variable on ITE. The SHAP results indicate that age, functional status measured at the ADLH scale, and the use of restraints were relevant variables in all models for predicting ITE. Figure 4 shows PDP plots for the six variables with the highest absolute sum of SHAP values from the causal forest model. The PDP plots present how variables affect predictions. The variables associated with the highest risk reduction in the probability of hospitalization with antipsychotic discontinuation, estimated by the causal forest model, were no use of restraints, better functional status (ADLH score), younger age, better cognitive status (CPS score), higher number of drugs, and hospital or emergency care visits. Surrogate model analyses present similar results as PDP plots (as illustrated in Additional file 1: Fig. S3).

### Model performance

**Fig. 5:**
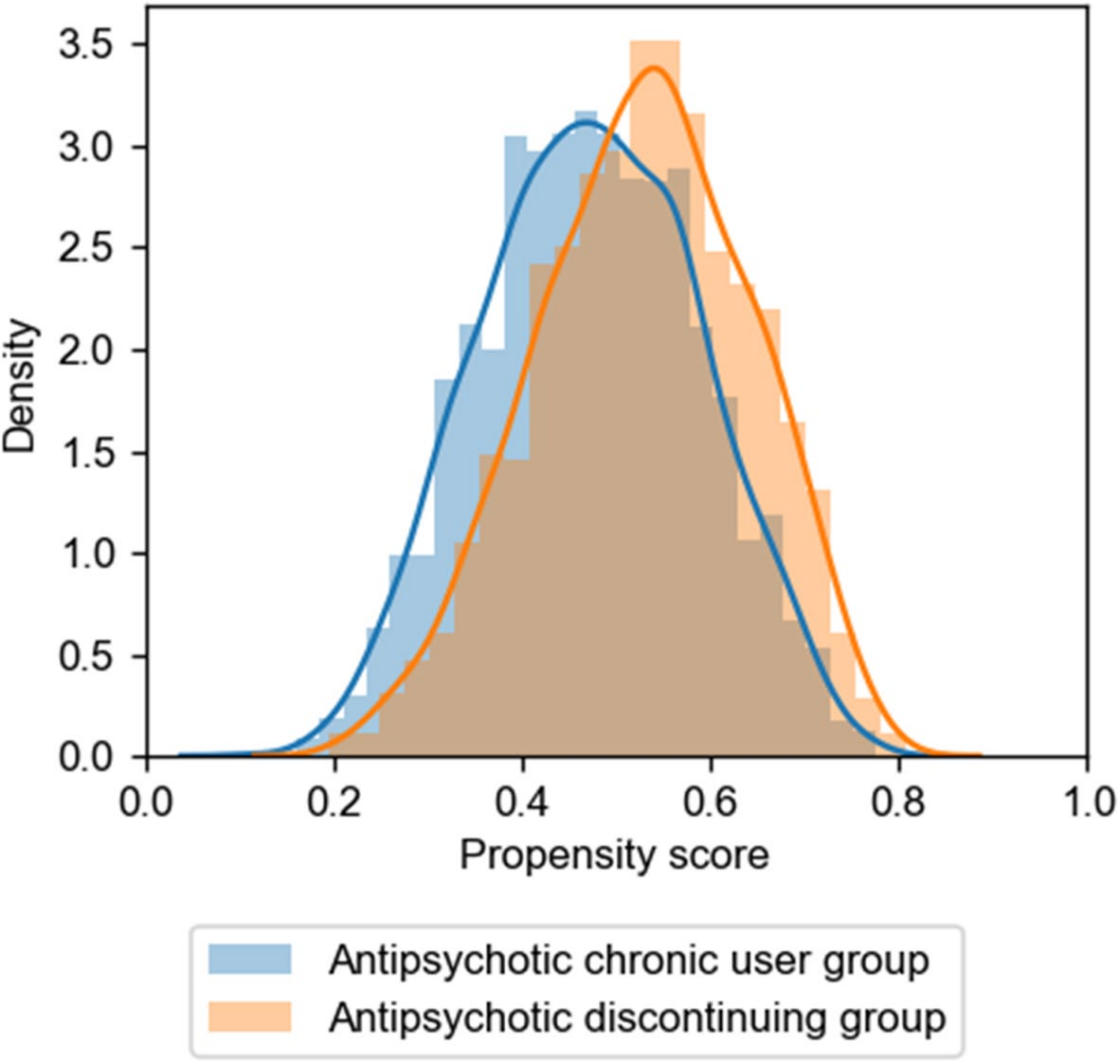
Propensity score distributions of antipsychotic discontinuing and chronic user groups

The predicted propensity scores showed no positivity violations. They were concentrated away from 0 and 1, with good overlap between the distributions for the discontinuing and chronic user group (as illustrated in Fig. 5). The imbalance of the inverse propensity weighted confounders was low. All confounders achieved good balance (as illustrated in Additional file 1: Fig. S4). The AUUC and c-for-benefit values of the null model are 0 and 0.5. Larger positive value indicates a better performance of the estimation algorithm for predicting treatment heterogeneity. All developed ITE models consistently outperformed the null model, demonstrating a modest but reliable ability to estimate patient-specific benefit from the discontinuation of antipsychotics. The observed AUUC values ranged from 0.038 to 0.063, and c-for-benefit values from 0.523 to 0.548, indicating meaningful improvements in performance compared to a random model. In particular, the causal forest model achieved the highest performance (AUUC = 0.063; c-for-benefit = 0.548). Although the AUUC and c-for-benefit values obtained in our study are numerically small, they are consistently positive across models and comparable to those reported in similar studies applying ITE models in clinical data [31, 38]. Furthermore, the high correlation of ITE estimates across different algorithms (ranging from 0.88 to 0.99) supports the reproducibility of the results and strengthens the trustworthiness of the estimated treatment heterogeneity. Results from correlation analyses are included in Additional file 1: Table S3.

### Sensitivity analyses

Sensitivity analyses and experts’ assessments supported the reliability of the modeling results (average ATE), which suggest that discontinuing antipsychotics is associated with a reduced risk of hospitalization. In a sensitivity analysis that measured the effect for modeling when train and test sets were randomly split, ATE, AUUC, and c-for-benefit values for test data were close to the values from the original analysis (Additional file 1: Table S4). In sensitivity analyses that measured the impact of randomly assigned treatment or outcome, ATE values for test data after re-training the models were close to zero (Additional file 1: Table S5). In a sensitivity analysis that measured the impact of adding a random confounder, ATE values for test data after re-training the models remained consistent with the original models (Additional file 1: Table S5).

## Discussion

In the present study, we have reported a mean decrease of 12% in the absolute probability of hospitalization for LTCF residents who stopped antipsychotic medications. Our findings indicate that among those residents who discontinue antipsychotics, over half of them will have a quite certain reduction in the risk of hospitalization, and this benefit is likely to extend to the majority of the population. Hospital admissions are frequent in long-term care. Most residents are frail older individuals with complex chronic conditions, requiring extensive assistance in the activities of daily living and with a certain degree of cognitive impairment. Because of their intrinsic vulnerability, LTCF residents are likely to experience sudden loss of health stability requiring acute care admission. It has been demonstrated that, for these individuals, hospitalizations often result in an increased burden of diseases and worsened quality life [39]. Additional morbidity related, for example, to the occurrence of delirium, pressure ulcers, deconditioning, weight loss, and falls during hospitalization, may indeed worsen the overall health status and level of disability of those who survive thus leading to further need of assistance and increased costs.

According to previous estimates, up to 67% of hospitalizations among LTCF residents might be preventable [39]. For example, promoting pneumococcal vaccination in LTCFs may reduce the incidence of pneumonia and related hospital admissions. Similarly, the correct and cautious management of medications is likely to reduce the probability of adverse drug reactions including serious effects requiring hospitalization.

Antipsychotic medications may increase the risk of hospitalization through several mechanisms. Most antipsychotic agents have the propensity to induce extrapyramidal syndrome including parkinsonism, gait disturbance, and rigidity, even at therapeutic dosages. These symptoms may eventually increase the risk of falling and developing swallowing disturbances with an ultimate increased risk of femur fracture and aspiration pneumonia [40]. Central and peripheral anticholinergic effects of antipsychotic medications are directly responsible of other relevant adverse events such as orthostatic hypotension, delirium, sedation, and dry mouth, conditions also predisposing to falls, malnutrition, and aspiration pneumonia [40]. Antipsychotic medications, especially second-generation agents such as risperidone, olanzapine, and quetiapine, have been also shown to increase the risk of deep venous thrombosis, pulmonary embolism, and acute cerebrovascular events [41]. Finally, many drugs including cardiovascular agents such as angiotensin-converting enzyme inhibitors, diuretics, angiotensin receptor blockers, calcium channel blockers and digoxin, several antibiotics, benzodiazepines, lithium, warfarin, and nonsteroidal antiinflammatory drugs share common metabolic pathways with antipsychotic medications, so that their concomitant use increases the probability of drug interactions and the risk of related adverse events [42].

The observed association between specific resident characteristics including younger age, better functional and cognitive status, no use of restraints, polypharmacy, high health care resource utilization, and a greater reduction in hospitalization risk after antipsychotic discontinuation deserve also some considerations. Younger age and better functional and cognitive status suggest higher compensatory capacity, enabling residents to better tolerate the withdrawal of antipsychotics without triggering events such as behavioral worsening, psychotic symptoms, or falls. Moreover, there is evidence that younger and better-functioning residents are indeed hospitalized more frequently [43]. This would be not necessarily due to worse health, but rather to a clinical attitude towards more active and aggressive care for these individuals in contrast to older, more frail residents. The greater effect of antipsychotic discontinuation in those who are younger and better preserved individuals may reflect their higher baseline hospitalization rate, which increases the absolute potential for risk reduction. No use of restraints likely reflects lower behavioral complexity, reducing the probability of adverse events following discontinuation. Polypharmacy was also associated with greater benefit, which may reflect the potential for reducing drug–drug interactions and the overall pharmacological burden. Lastly, high healthcare resource utilization could be interpreted as a proxy for clinical instability, elevated treatment burden, or active disease processes. In these cases, the benefit of antipsychotic discontinuation might stem from both reducing unnecessary pharmacological load and avoiding antipsychotic related adverse effects in multimorbid and clinically complex individuals.

The ML-based innovative methodology that has been adopted to answer the research question brings additional value to the evidence reported in this study. In clinical medicine, randomized control trials (RCTs) have been established as the gold standard method for investigating cause-and-effect relationships. However, due to the fixed characteristics of the experimental design, evidence from RCTs is rarely generalizable to older adults in the real world, especially to those individuals with frailty, multiple chronic diseases, and high vulnerability to drug interactions and adverse reactions such as the older LTCF residents. Large electronic health record systems and artificial intelligence (AI) based techniques offer an opportunity to estimate conditional average treatment effect directly from observational data through the adoption of causal ML methods. In other words, ML methods that are applied to large and high-quality observational data offer the possibility to make individual predictions taking into account the relative contribution of each factor influencing the outcome. This approach allows to estimate absolute risks and therefore it appears particularly promising for research in the geriatric field where, given the relevant heterogeneity of individuals, the knowledge of the individual risks may prove highly valuable in guiding clinical practice and making therapeutic decisions.

Study findings should be interpreted taking into account that further research is needed to replicate this evidence and support conclusions. Also, several study limitations need to be acknowledged. The performance of ITE models cannot be directly evaluated because the ground truth is not available. In this study, the performance of ITE models was evaluated by the similarity of the results from the different models and by running sensitivity analyses. Second, selection bias is a recurrent problem in observational research. In this study, we mitigated selection bias by selecting the confounders using domain expertise. We also tested that there was considerable overlap in the distributions of the propensity scores between the discontinuing and chronic user groups. In addition, propensity score distributions showed that the positivity assumption held, which is a requirement for causal inference. In the current analysis, we focused on all-cause hospitalization as the primary outcome, without formally modeling death as a competing risk (only those residents who were alive during the follow-up were included in the study). This choice may have limited the ability to fully disentangle the effects of antipsychotic discontinuation on the risk hospitalization and death. We considered only hospitalizations occurring during the 1-year follow-up period. Therefore, it is not possible to extend the observed effect of antipsychotic discontinuation to longer timeframes. Although cause-specific mortality was not assessed, this analysis may nonetheless offer valuable insights into the understanding of the relationship between antipsychotic discontinuation and the risk of hospitalization. Finally, the results of the present investigation refer to the specific setting and population studied and may not be generalizable to other groups of individuals.

## Conclusions

In summary, by applying innovative ML methods, we could show that to discontinue antipsychotic medications among older LTCF residents may contribute to the prevention of hospitalization. The risk reduction estimate obtained in this study also considered the relative contribution of numerous individual factors influencing the same outcome thus ultimately taking into account individual clinical heterogeneity. Antipsychotic discontinuation must be promoted by policies and public health programs to improve quality of life and reduce morbidity, mortality, and costs in long-term care.

## Supplementary Information

Additional file 1: Supplementary methods: additional details on data preprocessing, workflow of causal machine learning (ML) model training and evaluation, assumptions of causal modeling, confounder selection, individual treatment effect models, model evaluation, correlation matrix, performance of ML models for predicting factual outcomes, treatment effect distributions, heterogeneity, model interpretation, and sensitivity analyses. Fig. S1 Treatment definition. Two study groups were defined, treatment (antipsychotic discontinuing group) and control (antipsychotic chronic users) groups from the data of the long-term care residents who had at least four RAI assessments. Resident was classified in the treatment group if antipsychotics were prescribed at the baseline period (assessments 1 and 2) but no at the follow-up period (assessments 3 and 4). Resident was classified in the control group if antipsychotics were prescribed at the baseline and follow-up periods (assessments 1–4). The outcome of hospitalization was measured within 360 days of the first follow-up RAI assessment (assessment 3). The input variables in the models were collected from baseline (assessment 2) before treatment. For residents with more than one group of four RAI assessments, the assessment group was randomly selected from the valid groups. Fig. S2 Distributions of the estimated treatment effect values. Distributions of the estimated individual treatment effect (ITE) values of (a) X-learner, (b) double robust (DR) learner, (c) double machine learning (DML), and (d) causal forest. Fig. S3 Surrogate model of causal forest model. Surrogate decision tree derived from the causal forest model, illustrating key variables contributing to treatment effect heterogeneity. Fig. S4 Confounder balance of antipsychotic discontinuing and chronic user groups. Confounder balance between residents in the two study groups before and after inverse propensity weighting assessed with the absolute standardized mean difference (SMD). Confounders with absolute SMD below 0.1 are considered well balanced. Table S1 Variables that were processed from the source data. List of variables that were processed from the data source and univariate performance for predicting outcome (hospitalization) and treatment (antipsychotic discontinuation). Univariate performance was calculated by tenfold cross-validation of training data with logistic regression algorithm. Parameter “coef” indicates the direction of the effect. Table S2 Confounders. List of confounders that were selected by a group of three study researchers, experts from the field of clinical geriatrics and pharmacology. Table S3 Correlation values of the estimated treatment effects. Correlation values between the individual treatment effect estimates of double machine learning (DML), double robust (DR) learner, X-learner, and causal forest of test data. Table S4 Sensitivity analysis 1. Average treatment effect (ATE), area under uplift curve (AUUC), and c-for-benefit values for double machine learning (DML), double robust (DR) learner, X-learner, and causal forest models. Data randomly split for training and testing sets. Table S5 Sensitivity analysis 2. Average treatment effect (ATE) and 95% confidence intervals (CI) for double machine learning (DML), double robust (DR) learner, X-learner, and causal forest of test data when actual treatment variable or outcome was replaced with a random variable or a random confounder was added in the model.

## Data Availability

The data that support the findings of this study are available from Finnish Institute for Health and Welfare but restrictions apply to the availability of these data, which were used under license for the current study, and so are not publicly available. Data permits can be requested from the Finnish Social and Health Data Permit Authority, Findata. Please contact info@findata.fi for further information.

## Abbreviations

ABS: Aggressive behavior scale
ADLH: Activities of daily living hierarchy
BMI: Body mass index
CF: Causal forest
CPS: Cognitive performance scale
DML: Double machine learning
DR: Double robust
DRS: Depression rating scale
LTCFs: Long-term care facilities
ML: Machine learning
PDP: Partial dependence plots
RAI-LTC: Resident Assessment Instrument for Long-Term Care
SD: Standard deviation
SHAP: SHapley Additive exPlanations
