# Supplementary material for: Effect of discontinuing antipsychotic medications on the risk of hospitalization in long-term care: a machine learning-based analysis
Source: BMC Med. 2025 Aug 20;23:484. doi: 10.1186/s12916-025-04304-7 (PMC12366079; doi:10.1186/s12916-025-04304-7)
Supplement: Supplementary file 1 — Additional file 1: Supplementary methods: additional details on data preprocessing, workflow of causal machine learning (ML) model training and evaluation, assumptions of causal modeling, confounder selection, individual treatment effect models, model evaluation, correlation matrix, performance of ML models for predicting factual outcomes, treatment effect distributions, heterogeneity, model interpretation, and sensitivity analyses. Fig. S1 Treatment definition. Two study groups were defined, treatment (antipsychotic discontinuing group) and control (antipsychotic chronic users) groups from the data of the long-term care residents who had at least four RAI assessments. Resident was classified in the treatment group if antipsychotics were prescribed at the baseline period (assessments 1 and 2) but no at the follow-up period (assessments 3 and 4). Resident was classified in the control group if antipsychotics were prescribed at the baseline and follow-up periods (assessments 1–4). The outcome of hospitalization was measured within 360 days of the first follow-up RAI assessment (assessment 3). The input variables in the models were collected from baseline (assessment 2) before treatment. For residents with more than one group of four RAI assessments, the assessment group was randomly selected from the valid groups. Fig. S2 Distributions of the estimated treatment effect values. Distributions of the estimated individual treatment effect (ITE) values of (a) X-learner, (b) double robust (DR) learner, (c) double machine learning (DML), and (d) causal forest. Fig. S3 Surrogate model of causal forest model. Surrogate decision tree derived from the causal forest model, illustrating key variables contributing to treatment effect heterogeneity. Fig. S4 Confounder balance of antipsychotic discontinuing and chronic user groups. Confounder balance between residents in the two study groups before and after inverse propensity weighting assessed with the absolute standardized mean di [file 12916_2025_4304_MOESM1_ESM.pdf]

Effect of discontinuing antipsychotic medications on the risk of hospitalization in long term care: a machine learning based analysis

Additional File 1

Supplementary methods.
Figure S1. Treatment definition
Figure S2. Distributions of the estimated treatment effect values
Figure S3. Surrogate model of causal forest model
Figure S4. Confounder balance of antipsychotic discontinuing and chronic user groups
Table S1. Variables that were processed from the source data
Table S2. Confounders
Table S3. Correlation values of the estimated treatment effects
Table S4. Sensitivity analysis 1
Table S5. Sensitivity analysis 2

Supplementary Methods

Data preprocessing

The data sources are RAI-LTC (Resident Assessment Instrument for Long Term Care) assessments (MDS 2.0 instrument) and the Finnish Care Register for Health Care. The main task of preprocessing of data was the processing of categorical variables, missing values and outliers. Categorical nominal variables (e.g., gender, marital status) were encoded to binary variables. Categorical ordinal variables (e.g., education, cognitive skills) were encoded to continuous integer variables and binary variables. If background information was missing (gender, BMI and age), the resident was excluded from the study. For binary variables [0, 1], where missing values were assumed to mean positivity (e.g., no disease, no pain, no memory problem), the values were replaced with 0. For continuous or categorical ordinal variables missing values or outliers were replaced by the nearest neighbor imputation method.

Workflow of causal ML model training and evaluation

The workflow of causal machine learning (ML) model training and evaluation in this study is broken down into the three steps (Figure 1, main manuscript). The first step (phase 1) is to choose how generalizability of the models is measured, that is, what data is used for internal and external validation or what data is used for model training and testing. Second step (phase 2) evaluates the main assumptions required for causal modelling. Third step (phase 3) applies surrogate measures for evaluating internal and external validity of causal ML models.

In this study data was split for training and testing based on the index day (June 1st, 2016). Before any processing, data was divided in the ratio of 70% for training (before index date) and 30% for testing (after index date). All hyperparameters and base ML models were searched by k-fold (k=5) cross validation with the training data. After the models were trained by using training data, surrogate measures and model evaluations were applied for the test data.

Assumptions of causal modelling

The three principal assumptions of causal ML modelling are (1) SUTVA (stable unit treatment value assumption), (2) unconfoundedness (conditional exchangeability) and (3) positivity / overlap.[22]

SUTVA defines that potential outcomes for a given patient respond only to its own treatment status. That is, potential outcomes are invariant to treatment assignments of others.

Exchangeability means that the treatment and control groups are interchangeable in the sense that if they were switched, the new treatment group would observe the same outcomes as the old treatment group and the new control group would observe the same outcomes as the old control group. That is, the treatment and control

groups are the same in all relevant patient characteristics other than the treatment. However, in observational data it is unrealistic to assume that the treatment and control groups are interchangeable. We can expect that the characteristics of control and treatment groups differ. However, if we can control for all relevant variables (confounders), then control and treatment groups can be exchangeable. The unconfoundedness (conditional exchangeability) assumption means that all the variables affecting both the treatment and the outcome are measured and can be controlled for. It should be noted that unconfoundedness is an untestable assumption. If unmeasured confounding is present, the estimated treatment effects may suffer from confounding bias and can be incorrect.

Positivity is the condition that for each possible combinations of patient characteristics we can observe both treated and untreated patients. Violation of this assumption for individuals means that we cannot construct counterfactual for them. The positivity-unconfoundedness tradeoff means that increasing the number of controlled variables, can lead to a higher risk of violating positivity. By adding more controlled variables, the patient subgroups get smaller and the risk that whole subgroups will have only treatment or control patients increases.

Confounder selection

Because of unconfoundedness assumption and positivity-unconfoundedness tradeoff, the confounder selection is a fundamental phase of causal ML model development. In observational data analyses confounder selection is limited those variables that are previously measured in the database. Thus, the aim of confounder selection is to select the subset of available variables that minimizes error in the resulting treatment effect estimate. In this study, the confounders were selected from the 298 variables that were processed from the data sources (table S1). The variables include information of background, medications, disease diagnoses, health conditions, service utilization, memory, mood and behavior and activity of LTC residents. To select the most comprehensive set of confounders, we applied knowledge-based confounder selection approach. The group of three experts, informed by the data-driven result (see the data-driven results from the AUROC columns of table S1), formed a unified opinion on the set of confounders for the causal ML models. The aim was to select variables that are cause of the treatment or outcome or both, then, additionally, discarding any variable known to be an instrumental variable, and including variables that do not satisfy the criterion but are good proxies for unmeasured common causes of the treatment and the outcome. The descriptions of the selected confounders are presented in table S2. Figure 5 in main manuscript shows propensity score distributions of chronic users (identified as control group) and previous users (those who discontinued antipsychotics, identified as treatment group) for the training data. The propensity scores modelled from the selected confounders shows substantially overlap between the control and treatment groups, and the propensity scores are concentrated away from 0 and 1, that are requirements for causal ML models. The absolute standardized mean difference (SMD) of the unadjusted and inverse follow

weighted (IPW) confounders between the treatment and control groups are reported in figure S4. Confounders after IPW are well balanced (SMD < 0.1).

Individual treatment effect models

Currently, there is no generally accepted standard algorithm for estimating individual treatment effect (ITE). So in this study we used several different algorithms (X-learner, DML, DR-learner and causal forest) and compared their performance to each other.

X-learner estimates treatment effects $\hat{\tau}$ by the equations:

$$\widehat{D}1(X) = Y(1) - \hat{u}0(X) \; for \; T = 1 \quad (2)$$

$$\widehat{D}0(X) = \hat{u}1(X) - Y(0) \; for \; T = 0 \quad (3)$$

$$\hat{\tau}(X) = \hat{g}(X)\hat{\tau}0(X) + [1 - \hat{g}(X)]\hat{\tau}1(X) \quad (4)$$

where $Y(1)$ and $Y(0)$ are observed outcomes for treated and control, $\hat{u}1(X)$ and $\hat{u}0(X)$ are outcome prediction models for treated and control, $\hat{\tau}1(X)$ and $\hat{\tau}0(X)$ are treatment effect prediction models for treated and control trained by $\widehat{D}0$ and $\widehat{D}1$, $\hat{g}(X)$ is propensity score model and X are the covariates characterizing the individuals. The estimated treatment effects $\hat{\tau}$ can then be used for training ITE θ model. The expressions $\hat{u}(\cdot)$ and $\hat{g}(\cdot)$ are often referred as nuisance functions or base learners, and they can be estimated with any machine learning algorithms. In this study, we applied random forest, logistic regression, and gradient boosting algorithms. The base learners of each causal ML model and hyperparameters were selected based on k-fold cross-validation from the training data.

Double machine learning (DML) is estimated by the equations (5-8). The patients are divided into K subsamples, then $\hat{u}(X)$ and $\hat{g}(X)$ are estimated in each subsample and residuals-on-residuals regression models are formed (Eq. 5).

$$\left(Y - \hat{u}(X)\right) = \theta(X)(T - \hat{g}(X)) \quad (5)$$

The score function φ is formed as a dot product of the error terms of residuals-on-residuals regression and the propensity score model $\hat{g}(X)$:

$$\varphi\big(Z;\theta,h(X)\big) = (Y - \hat{u}(X) - \theta(X)(T - \hat{g}(X)) \cdot (T - \hat{g}(X))$$
(6)

where $Z = \{Y, T, X\}$ are the observed variables and $h = \{u(X), g(X)\}$ are prediction models. The treatment effect estimator is constructed as the solution to:

$$\frac{1}{K}\sum_{k=1}^{K}\sum_{i=1}^{n}\varphi\left(Z_i;\theta,\hat{h}(X_i)\right) = 0$$
(7)

Estimated $\hat{\theta}$ minimizes the average of score functions over all K subsamples.

Double robust (DR) learner estimates treatment effects $\hat{\tau}$ by the equation:

$$\hat{\tau}(X) = \hat{u}1(X) - \hat{u}0(X) + \frac{T*\big(Y-\hat{u}1(X)\big)}{\hat{g}(X)} - \frac{(1-T)*(Y-\hat{u}0(X))}{(1-\hat{g}(X))}$$
(8)

and the estimated treatment effects $\hat{\tau}$ can then be used for training ITE θ model.

Causal forests are special cases of generalized random forest.[23] Compared to random forests, causal forests use different function to split the nodes. Splitting the data in the training phase of causal tree is based on calculating equation (5) for each candidate split. The aim of splits is that the estimated treatment effects are similar within a node, but differ as much as possible between different nodes. This is performed on many bootstrap samples, thus forming a causal forest. In the treatment effect estimation phase, the samples are fitted to causal trees to determine end nodes of the sample. Data-derived weights a are calculated for the training samples, which are derived from the frequencies of the neighborhood of the test sample in the different end nodes of causal trees. ITE θ is estimated by solving the weighted moment equation using these data-derived weights and the score function φ:

$$\sum_{i=1}^{n} a_i(X)\varphi\left(Z_i;\theta,\hat{h}(X_i)\right) = 0$$
(9)

Model evaluation

The fundamental problem with causal ML model evaluation is that the accuracy of causal ML models cannot be measured directly by comparing predicted and true outcome values in test set as can be done with conventional ML models. Instead, causal ML model evaluation should be based on a set of surrogate measures

and expert evaluations. Surrogate measures present information about functionality of the models and what models have learned from data. Although surrogate measurements give numerical values, clinical experts are needed for interpretating results and forming an opinion on whether the results can be trusted.

Correlation matrix: Correlation matrix of individual treatment effect values from different causal ML models is used for evaluating the reproducibility of estimates.[24] The aim is to measure if similar positive/negative/neutral effects between treated and control patients have been found with different algorithmic solutions. The hypothesis is that similar estimates (high correlation) with different algorithms increase the trustworthy of results. Correlation values between the ITE estimates of DML, DR-learner, X-learner and Causal forest of the test data are reported in table S3. Correlation values are between 0.88 and 0.99, which means that different algorithms of this study estimated similar patient-specific treatment effect estimates that increase the trustworthy of the results.

Performance of base ML models for predicting factual outcomes: One method of evaluating causal ML method is to evaluate the accuracy in predicting factual outcomes.[25] This can give some insight into whether the disease mechanism is captured. In this study, the accuracy of the factual outcome predictions was used for selecting the base learners and the hyperparameters of the causal ML models. However, the fundamental limitation is that treatment effects cannot be evaluated because counterfactual results cannot be observed.

Treatment effect distributions: Based on the average treatment effect (ATE), the average effect of treatment is positive, negative, or neutral. Even if ATE is neutral, some patients can have positive or negative individual treatment effect (ITE). In this study, the distributions of ITE values were analyzed with histograms and sorted values. Histograms of the estimated ITE values of X learner, DR learner, DML, and causal forest are illustrated in figure S2.

Heterogeneity: If model found heterogeneity from data, then model-assisted recommendations are better than random treatment assignment. In this study we used the measures of area under uplift curve (AUUC) and Concordance-statistics for benefit (c-for-benefit) for evaluating heterogeneity of the estimates. AUUC is calculated as the cumulative difference between the baseline and the uplift curve values of model. For calculating uplift curve for model, the test set samples are sorted by rank of estimated ITE values, and subsamples consisting of top k samples are created for each value of k (k = 10, 20, ..., n; n = total sample size). The uplift curve plots the estimated difference in outcome events (e.g. hospitalization) between treated and control groups, calculated from observed outcomes in each subsample. The baseline curve is calculated by random ITE values. C-for-benefit measures the probability that from two randomly chosen matched patient pairs with unequal observed benefit, the pair with greater observed benefit also has a higher predicted benefit. Matching of treated and control patients can be based on predicted treatment effect or variables that are used to predict treatment effect.

Model interpretation

For interpretation of the ITE models, we used the methods of SHAP values (SHapley Additive exPlanations), partial dependence plots (PDP), and surrogate models.

Shapley values are sample specific variable importance (magnitude and sign of contribution) estimates. Variables with positive Shapley estimates contribute to the prediction of positive outcome, whereas variables with negative estimates contribute to the prediction of negative outcome. The Shapley value of a variable i is defined by: $\emptyset_i = \frac{1}{|N|!}\sum_{S\subseteq N\{i\}}|S|!\,(|N|-|S|-1)!\,[f(S\cup\{i\})-f(S)]$ where $f(S)$ is the outcome of model using a set S of variables, and N is the complete set of all variables. The contribution of variable i (Shapley value, $\emptyset_i$) is calculated as the average of its contributions across all possible permutations of a variable set.

Partial dependence plots (PDP) show the dependence between predicted outcome (e.g. individual treatment effect) and a set of input variables of interest (such as BMI, age, ADL), marginalizing over the values of all other input variables. The partial function $g_s(x_s)$ is estimated by calculating averages in training data: $g_s(x_s) = \frac{1}{n}\sum_{i=1}^{n} g(x_s, x_C^{(i)})$ where x_s is the variable for which the partial function is calculated, x_C are other variables used in model $g()$ and n is the number of samples. By marginalizing over the x_s, partial function g_s depends only on variable x_s, but including interactions with other variables x_C. PDP is a method for analysing correlation structure that model learned from training data. PDP can show whether the relationship between the outcome and a variable is linear, monotonic or more complex.

Surrogate model is an interpretable model that is trained to approximate the predictions of a complex model . That is, the goal of surrogate model $g(X)$ is to approximate the predictions of underlying complex model $f(X)$ as accurately as possible and to be interpretable at the same time. For the function g any interpretable model (such as linear model or decision tree) can be used. Training of $g(X)$ requires input variables X and predicted outcomes from $f(X)$. For example, surrogate tree can be used for presenting the most important variables and the thresholds of the variables of model $g(X)$. A surrogate model of causal forest model is illustrated in figure S3.

Sensitivity analyses

To validate the robustness of the treatment effect estimates against explicit violations of the different assumptions, different sensitivity analyses should be conducted.[26] One method is to add a random variable to check if the treatment effect estimates remain consistent (as such a variable should not affect the estimates). Another method is to replace the actual treatment variable (or outcome variable) with a random variable to check if the estimated treatment effect goes to zero. ATE, AUUC and c-for-benefit values for double machine

learning (DML), double robust (DR) learner, X learner and causal forest models when data are randomly split for training and testing sets are reported in table S4.
ATE and 95% CI values for the test data when the models were trained with a random treatment effect, random outcome or random variable was added in the set of confounders are reported in table S5 .

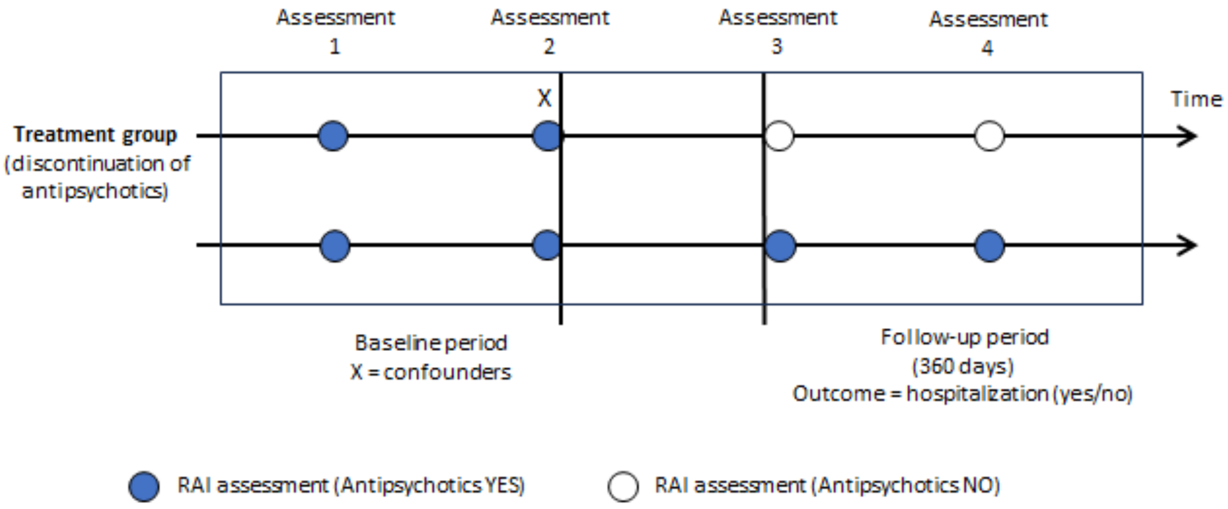

Figure S1. Treatment definition.

Two study groups were defined, treatment (antipsychotic discontinuing group) and control (antipsychotic chronic users) groups from the data of the long-term care residents who had at least four RAI assessments. Resident was classified in the treatment group if antipsychotics were prescribed at the baseline period (assessments 1 and 2) but no at the follow-up period (assessments 3 and 4). Resident was classified in the control group if antipsychotics were prescribed at the baseline and follow-up periods (assessments 1-4). The outcome of hospitalization was measured within 360 days of the first follow-up RAI assessment (assessment 3). The input variables in the models were collected from baseline (assessment 2) before treatment. For residents with more than one group of four RAI assessments, the assessment group was randomly selected from the valid groups.

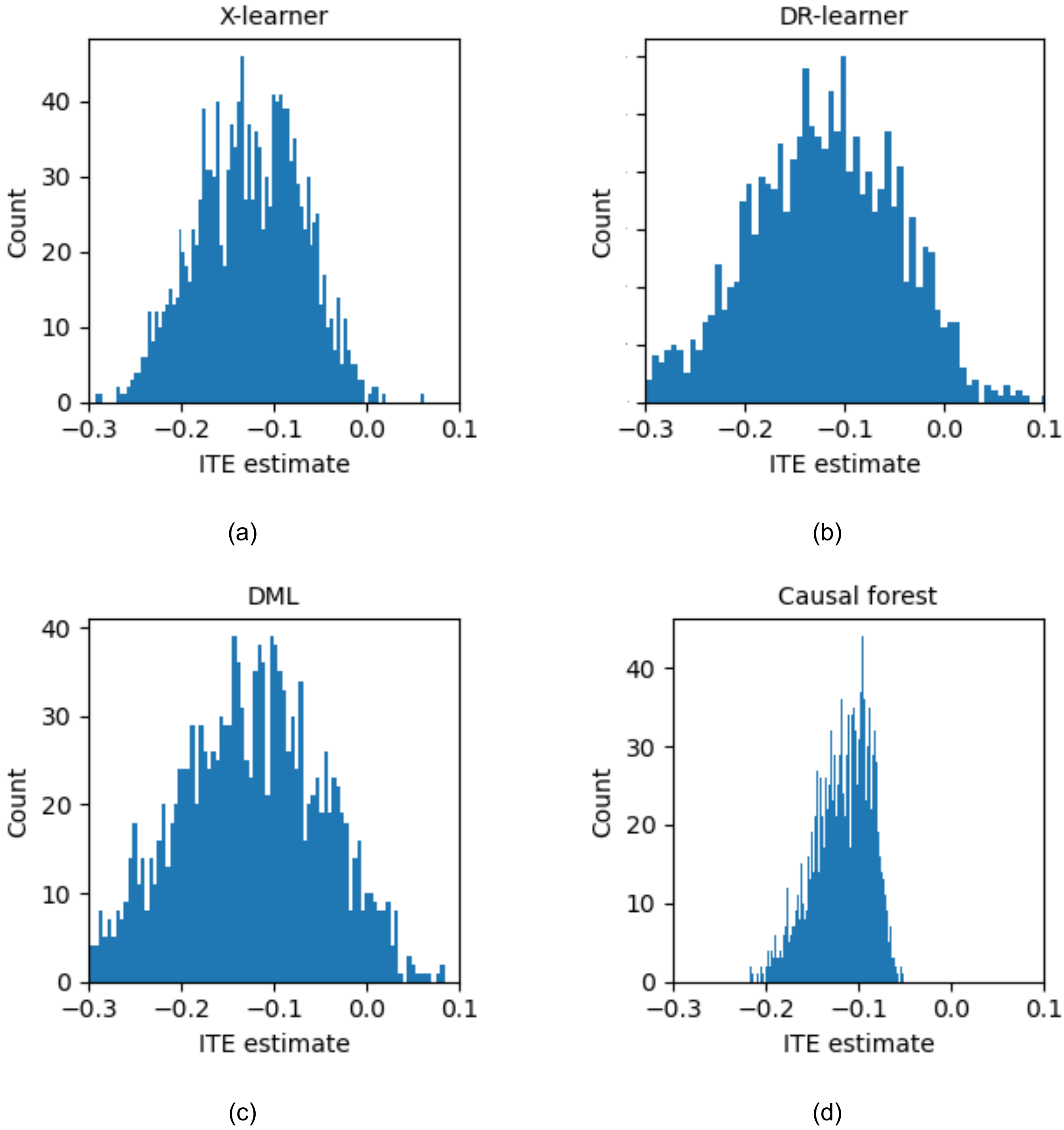

(a) (b) (c) (d)

Figure S2. Distributions of the estimated treatment effect values.

Distributions of the estimated individual treatment effect (ITE) values of (a) X -learner (b) double robust (DR) learner, (c) double machine learning (DML), and (d) causal forest.

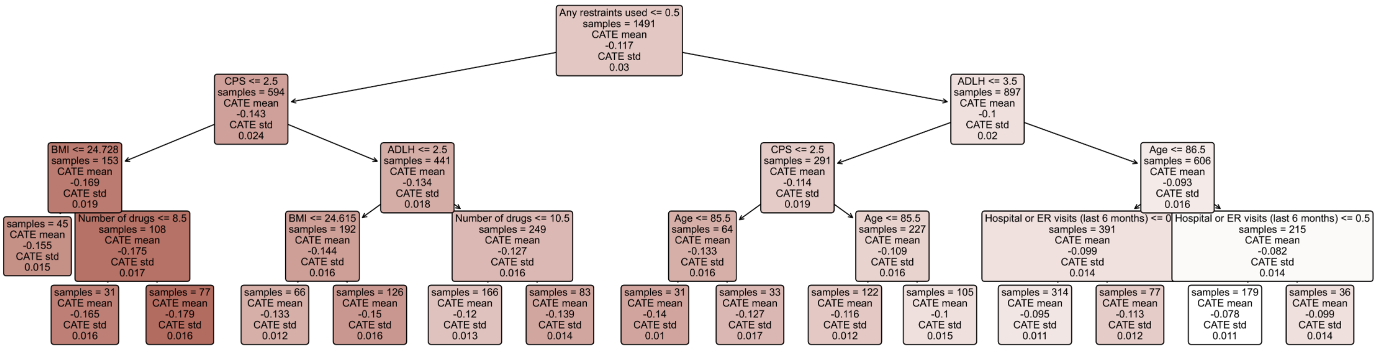

Figure S3. Surrogate model of causal forest model.

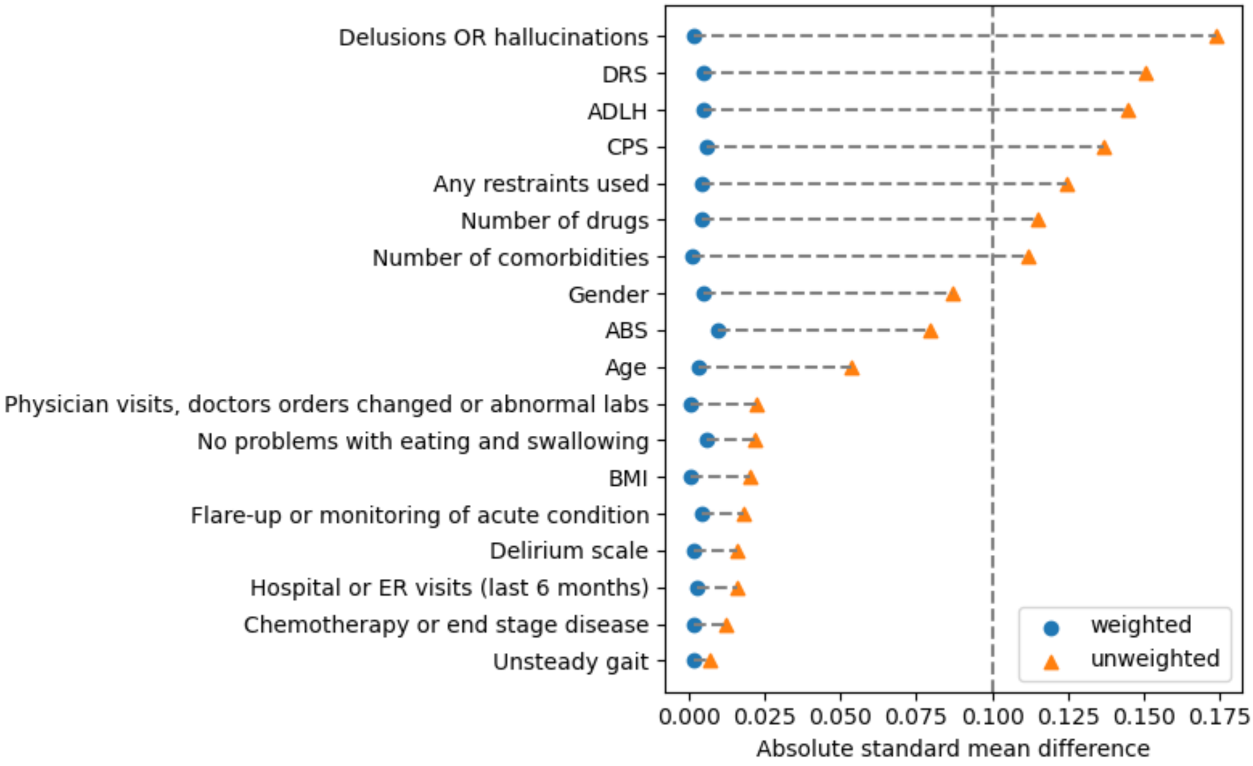

Figure S4. Confounder balance of antipsychotic discontinuing and chronic user groups.
Confounder balance between residents in the two study groups before and after inverse propensity weighting assessed with the absolute standardized mean difference (SMD). Confounders with absolute SMD below 0.1 are considered well balanced.

Table S1. Variables that were processed from the source data.

List of variables that were processed from the data source and univariate performance for predicting outcome (hospitalization) and treatment (antipsychotic discontinuation). Univariate performance was calculated by 10-fold cross-validation of training data with logistic regression algorithm. Parameter "coef" indicates the direction of the effect.

Variable	Outcome (AUROC)	Outcome (coef)	Treatment (AUROC)	Treatment (coef)
ADLLF	0.592	-0.861	0.563	0.696
CPS	0.582	-1.120	0.560	0.946
ADLH	0.582	-0.888	0.552	0.682
Number of drugs	0.575	1.686	0.533	-0.769
Self performance - toilet	0.571	-0.430	0.539	0.346
Memory recall - location of own room	0.569	0.526	0.573	-0.577
Full bed rails on all sides	0.568	-0.455	0.547	0.355
Memory recall - current season	0.564	0.524	0.557	-0.469
Self performance - dressing	0.564	-0.538	0.531	0.292
Self performance - transfer	0.564	-0.553	0.525	0.236
Self performance - personal hygiene	0.563	-0.441	0.526	0.270
Cognitive skills for making every day decisions	0.562	-0.783	0.541	0.675
Memory recall - that he / she is in a facility	0.558	0.446	0.564	-0.550
Self performance - locomotion on unit	0.557	-0.500	0.526	0.232
Self performance - walk in room	0.557	-0.451	0.525	0.180
Memory recall - Nothing	0.556	-0.527	0.560	0.602
Self performance - bed mobility	0.555	-0.605	0.527	0.320
Long term memory	0.553	-0.519	0.530	0.434
Self performance - walk in corridor	0.547	-0.393	0.523	0.177
Hospital visits, last 6 months (HILMO)	0.547	3.596	0.503	-0.697
Anticoagulants	0.547	0.376	0.496	0.026
Antithrombotic agents	0.547	0.376	0.496	0.026
ER visits, last 6 months (HILMO)	0.547	2.365	0.495	0.176
DRS	0.544	0.698	0.550	-0.862
Number of comorbidities	0.542	0.874	0.543	-0.941
BMI	0.542	1.276	0.526	-0.780
Hospital stays in last 90 days	0.542	1.830	0.511	-0.662
Delirium - periods of restlessness	0.541	-0.312	0.522	0.019
Hospital LoS, last 6 months (HILMO)	0.540	1.780	0.501	-0.478
Short term memory	0.539	-0.558	0.534	0.674
Self performance - locomotion off unit	0.539	-0.272	0.514	0.114

Self performance - eating	0.538	-0.849	0.523	0.424
Repetitive anxious complaints or concerns (3 cat)	0.537	0.426	0.548	-0.580
Emergency room visits in last 90 days	0.537	0.669	0.501	0.680
Trunk restraint	0.536	-0.485	0.532	0.332
Diuretics	0.536	0.340	0.501	-0.061
Statins	0.535	0.322	0.497	-0.018
Hmg coa reductase inhibitors	0.535	0.322	0.497	-0.018
Unsteady gait	0.535	0.163	0.520	-0.062
Delusions	0.535	0.143	0.553	-0.604
Delirium - periods of lethargy	0.534	-0.228	0.530	0.246
Rehabilitation - eating or swallowing	0.534	-0.404	0.511	0.177
Antithrombotic agents vitamin k antagonists	0.534	0.647	0.493	0.014
Gender, female	0.534	-0.295	0.523	0.211
Repetitive health complaints (3 cat)	0.533	0.495	0.530	-0.772
Delirium - periods of altered perception or awareness of surroundings	0.533	-0.298	0.524	0.206
Hospital visits (unplanned), last 6 months (HILMO)	0.533	2.401	0.504	-0.477
Physician visits in last 14 days	0.533	1.088	0.521	1.125
Age	0.533	-0.265	0.526	0.292
Hospital LoS (unplanned), last 6 months (HILMO)	0.532	0.970	0.503	-0.236
Hypoglycemic medication	0.531	0.442	0.510	-0.210
Drug used in diabetes	0.531	0.442	0.510	-0.210
Beta blocking	0.531	0.315	0.506	-0.119
resists care - density	0.529	-0.175	0.494	0.051
Memory recall - staff names / faces	0.528	0.248	0.534	-0.324
Repetitive physical movements (3 cat)	0.528	-0.187	0.510	0.086
Heart failure medication	0.528	0.261	0.503	-0.090
physically abusive symptoms - density	0.528	-0.304	0.492	0.045
Diabetes mellitus	0.527	0.311	0.505	-0.114
Preferred activity - walking or wheeling outdoors	0.526	0.101	0.518	0.088
Agents acting on the ra system	0.526	0.202	0.488	-0.010
Acute episode or flare-up of recurrent or chronic problem	0.525	0.473	0.503	-0.188
Alzheimer	0.524	-0.248	0.575	0.488
Preferred activity - talking or conversing	0.524	0.214	0.516	-0.170
Chair prevents rising	0.524	-0.628	0.511	0.249
physically abusive symptoms - chenge	0.524	-0.190	0.495	0.034
no mouth problems	0.524	0.151	0.511	0.029

Negative statements of depression, anxiety and sad mood (3 cat)	0.523	0.280	0.514	-0.451
resists care - change	0.523	-0.103	0.504	0.040
Monitoring acute medical condition	0.523	0.487	0.517	0.241
Dizziness or vertigo	0.522	0.240	0.492	-0.053
Delirium - easily distracted	0.522	-0.154	0.502	0.051
Other types of bed rails	0.522	0.074	0.490	0.010
Rehabilitation - range of motion passive	0.521	-0.244	0.516	0.218
Delirium - episodes of disordered speech	0.521	-0.217	0.533	0.029
Expressions of what appear to be unrealistic fears (3 cat)	0.520	0.334	0.527	-0.420
One or more indicators of depression, anxiety or sad mood (3 cat)	0.520	0.103	0.523	-0.247
Emphysema or COPD	0.520	0.765	0.507	-0.377
Antithrombotic agents platelet aggregation	0.520	0.178	0.503	0.130
Self deprecation (3 cat)	0.519	0.500	0.520	-0.484
Lower education	0.518	0.187	0.514	-0.165
Preferred activity - spiritual or religious activities	0.518	0.177	0.510	-0.095
Cardiac dysrhythmia	0.518	0.307	0.504	-0.070
Fell in past 30 days	0.517	0.361	0.512	0.208
Average time involved in activities	0.517	-0.190	0.510	-0.100
Edema	0.517	0.233	0.508	-0.152
Crying, tearfulness (3 cat)	0.517	0.251	0.507	-0.136
Abnormal lab values in last 90 days	0.517	0.339	0.511	0.124
Schizophrenia	0.516	0.269	0.552	-1.245
Recurrent statements that something terrible is about to happen (3 cat)	0.516	0.397	0.520	-0.592
Repetitive questions of depression, anxiety and sad mood (3 cat)	0.516	0.146	0.517	-0.243
Preferred activity - trips or shopping	0.516	0.227	0.512	-0.215
Preferred activity - cards, other games	0.516	0.299	0.510	-0.186
No nutrition complaints	0.515	-0.183	0.535	0.293
Socially inappropriate symptons - density	0.515	-0.094	0.525	-0.250
Rehabilitation - walking	0.515	0.054	0.512	0.170
Antidepressants	0.515	0.060	0.487	0.016
Anxiolytics	0.514	0.074	0.527	-0.237
Insomnia or change in usual sleep pattern (3 cat)	0.514	0.316	0.522	-0.235
Fell in past 31 to 180 days	0.514	0.152	0.491	-0.002
Rehabilitation - bed mobility	0.513	-0.190	0.507	0.118

Days doctor orders changed in last 14 days	0.513	1.371	0.507	0.291
Intensity of pain in last 7 days	0.513	0.086	0.495	0.024
verbally abusive symptoms - density	0.512	0.137	0.539	-0.370
Insufficient fluid consumed in last 3 days	0.512	-0.184	0.498	0.041
Chemotherapy	0.512	0.516	0.500	-0.613
Anxiolytics benzodiazepine	0.511	0.065	0.527	-0.237
Preferred activity setting - day or activity room	0.511	0.107	0.513	0.149
Antithrombotic agents direct factor xa	0.511	-0.341	0.505	-0.283
Rehabilitation - other	0.511	0.132	0.505	-0.071
Hypnotics and sedatives benzodiazepine rel drugs	0.511	0.224	0.495	0.060
Sad, pained, worried facial expression (3 cat)	0.511	0.052	0.488	0.020
Limb restraint	0.511	-0.232	0.503	0.454
Anticholinergic medication	0.510	0.568	0.510	-0.750
Preferred activity - watching TV	0.510	0.186	0.508	-0.123
Headache	0.510	0.111	0.506	-0.133
Hypnotics and sedatives	0.510	0.086	0.503	-0.052
Preferred activity - crafts or arts	0.510	0.173	0.500	-0.053
Antidepressants SSRI	0.510	0.254	0.498	-0.010
Antidepressants SSRI_v2	0.510	0.254	0.498	-0.010
Arteriosclerotic heart	0.510	0.089	0.491	0.004
Anxiety disorder	0.509	0.206	0.516	-0.497
Shortness of breath	0.509	0.331	0.512	-0.373
Cardiac therapy	0.509	0.111	0.499	0.035
Glaucoma	0.509	-0.550	0.493	-0.001
End stage disease, 6 months or less to live	0.509	-0.717	0.500	0.365
Alzheimers or dementia special care unit	0.508	-0.070	0.523	0.211
ABS	0.508	-0.147	0.522	-0.254
Hypnotics and sedatives benzodiazepine	0.508	0.162	0.512	-0.317
Peripheral vascular	0.508	0.357	0.504	-0.222
changes in behavioural symptoms	0.508	0.076	0.497	-0.120
swallowing problem	0.508	-0.190	0.496	0.018
Rehabilitation - transfer	0.507	-0.050	0.514	0.136
Change in mood (3 cat)	0.507	0.118	0.509	-0.096
wandering - density	0.507	0.124	0.508	-0.025
Bipolar disorder, manic depressive	0.507	0.404	0.507	-0.714
Asthma	0.507	0.108	0.507	-0.193
Anemia	0.507	0.319	0.502	-0.119
Memory galantamin	0.507	0.448	0.500	0.310
Rehabilitation - splint or brace assistance	0.507	0.363	0.498	-0.097

Inability to lie flat due to shortness of breath	0.507	0.382	0.497	-0.040
Osteoporosis	0.507	0.162	0.496	0.099
Parkinson disease	0.507	0.186	0.495	-0.004
wandering - change	0.507	0.120	0.493	-0.004
Rehabilitation - range of motion active	0.507	-0.110	0.491	-0.018
Persistent anger with self or others (3 cat)	0.506	0.112	0.532	-0.193
Reduced social interaction (3 cat)	0.506	-0.055	0.514	-0.081
Liver disease	0.506	0.131	0.496	-0.048
Other cardiovascular	0.505	0.211	0.510	-0.136
Antidepressants mirtazapine	0.505	0.023	0.504	0.130
Anticholinergic agents	0.505	0.865	0.504	-1.254
Cerebrovascular accident	0.505	0.247	0.504	-0.046
Respiratory infection	0.505	-0.825	0.502	-0.424
Transient ischemic attack	0.505	0.282	0.501	0.243
Anti-inflammatory and antirheumatic products	0.505	-0.204	0.500	-0.152
Vomiting	0.505	0.355	0.498	0.165
Withdrawal from activities of interest (3 cat)	0.504	-0.043	0.517	-0.076
Pain medication	0.504	-0.028	0.506	0.030
Preferred activity - gardening or plants	0.504	-0.086	0.506	0.035
Aphasia	0.504	-0.121	0.504	-0.235
Evaluation by licensed mental health specialist in last 90 days	0.504	0.404	0.503	-0.281
Anxiolytics diphenylmethane	0.504	0.876	0.502	-0.305
Pathological bone fracture	0.504	0.662	0.499	-0.348
Living alone	0.503	0.179	0.520	-0.191
Depression	0.503	0.112	0.515	-0.234
Anticholinergics	0.503	0.358	0.506	-0.738
Seizure disorder	0.503	0.225	0.505	-0.142
Chest pain	0.503	0.455	0.504	-0.385
Preferred activity - exercise or sports	0.503	0.030	0.489	0.012
Other pain	0.502	0.089	0.513	-0.100
Rehabilitation - amputation or prosthesis care	0.502	-0.809	0.503	-0.752
Syncope	0.502	0.425	0.502	0.326
Days occupation therapy	0.502	-0.942	0.502	-0.359
Synthetic anticholinergics agents in combination with analgesics	0.502	0.517	0.500	-0.311
Renal dialysis	0.502	0.556	0.500	-0.284
Ostomy care	0.502	0.401	0.500	-0.689
Group therapy	0.502	-0.607	0.500	0.280

Antidepressants TCA	0.502	0.640	0.499	0.077
Antidepressants NSRI	0.502	0.640	0.499	0.077
Hyperthyroidism	0.502	0.317	0.499	0.150
Reorientation	0.502	0.343	0.499	0.085
Fibrates	0.502	0.666	0.498	0.041
Soft tissue pain	0.502	0.238	0.497	-0.106
Rehabilitation - dressing or grooming	0.502	-0.049	0.486	0.013
socially inappropriate symptons - change	0.501	-0.037	0.519	-0.207
Frequency of complaints or evidence of pain in last 7 days	0.501	0.086	0.508	-0.092
Cognitive, ADL, mood or behaviour patterns unstable	0.501	0.068	0.507	0.083
Antidepressants SNRI	0.501	-0.179	0.505	-0.251
Hip pain	0.501	0.096	0.504	-0.110
Antithrombotic agents other	0.501	0.125	0.501	-0.458
Oxygen therapy	0.501	0.918	0.501	-0.575
Cerebral palsy	0.501	-1.057	0.500	-0.201
Hemiplegia or hemiparesis	0.501	0.252	0.500	-0.043
Multiple sclerosis	0.501	-0.730	0.500	0.426
Diabetic retinopathy	0.501	0.439	0.500	-0.397
Conjunctivitis	0.501	0.774	0.500	0.126
Antithrombotic agents direct thrombin	0.501	0.486	0.499	-0.146
Pneumonia	0.501	0.627	0.499	-0.381
Ventilator or respirator	0.501	0.518	0.499	0.109
Respite care	0.501	0.298	0.499	0.097
Mins respiratory therapy	0.501	-0.543	0.499	-0.218
Missing limb	0.501	-0.348	0.497	-0.111
Fever	0.500	-0.037	0.501	0.426
Intake or output	0.500	0.277	0.501	-0.439
Mins psychological therapy	0.500	0.589	0.501	-0.564
Psychotropic medication	0.500	0.000	0.500	0.000
Antidepressants MAOIs	0.500	0.000	0.500	0.000
Antidepressants MAO_A	0.500	-0.421	0.500	-0.461
Anxiolytics carbamates	0.500	0.000	0.500	0.000
Anxiolytics dibenzo bicyclo octadiene	0.500	0.000	0.500	0.000
Anxiolytics other	0.500	0.000	0.500	0.000
Hypnotics and sedatives barbiturates plain	0.500	0.000	0.500	0.000
Hypnotics and sedatives barbiturates comb	0.500	0.000	0.500	0.000
Hypnotics and sedatives aldehydes	0.500	0.000	0.500	0.000
Hypnotics and sedatives piperidinedione	0.500	0.000	0.500	0.000

Hypnotics and sedatives other	0.500	-0.260	0.500	0.534
Synthetic anticholinergics quaternary ammonium compounds	0.500	-0.208	0.500	0.475
Cellulitis	0.500	-0.693	0.500	0.433
Sexually transmitted disease	0.500	-0.234	0.500	0.594
Tuberculosis	0.500	-0.996	0.500	-0.550
Weight gain or loss of 1.5+ kilos	0.500	0.483	0.500	-0.171
Internal bleeding	0.500	-0.437	0.500	-0.205
Recurrent lung aspirations in last 90 days	0.500	0.226	0.500	-0.133
Incisional pain	0.500	-0.957	0.500	0.146
Tracheostomy care	0.500	-0.355	0.500	-0.439
Hospice care	0.500	0.476	0.500	-0.602
Days respiratory therapy	0.500	-0.625	0.500	0.134
Coma or persistent vegetative state	0.500	-0.260	0.500	0.534
Clostridium difficile	0.500	-0.395	0.499	0.004
Training in skills required to return to community	0.500	-0.543	0.499	0.146
Mins speech therapy	0.500	0.110	0.498	0.113
Special behaviour symptom evaluation	0.500	-0.751	0.498	0.055
Resident specific deliberate changes in environment to address mood or behaviour patterns	0.500	-0.209	0.497	-0.034
Preferred activity - reading or writing	0.499	0.051	0.510	-0.120
Bone pain	0.499	-0.134	0.503	-0.288
Dehydrated, output exceeds input	0.499	-0.033	0.502	-0.222
Bile acid sequestrants	0.499	-0.219	0.501	0.761
Transfusions	0.499	0.200	0.501	-0.632
Days speech therapy	0.499	-0.152	0.501	-0.452
Mins occupation therapy	0.499	-0.025	0.501	-0.029
Antidepressants agomelatine	0.499	-0.161	0.500	-0.258
Anxiolytics azaspirodecanedione	0.499	-0.162	0.500	-0.301
Other lipid modifying agents	0.499	-0.214	0.500	0.189
Synthetic anticholinergics agents in combination with psycholeptics	0.499	0.396	0.500	0.078
Paraplegia	0.499	-0.026	0.500	-0.165
Macular degeneration	0.499	-0.421	0.500	0.168
Radiation	0.499	-0.046	0.500	-0.105
Suctioning	0.499	-0.221	0.500	-0.623
Alcohol or drug treatment program	0.499	0.114	0.500	0.425
Pediatric unit	0.499	-0.055	0.500	0.471
Septicemia	0.499	0.196	0.499	-0.087

Arthritis	0.499	-0.231	0.498	0.060
Stomach pain	0.499	0.123	0.495	-0.008
Memory rivastigmine	0.498	0.063	0.514	0.356
Traumatic brain injury	0.498	0.102	0.506	-0.384
Analgesics	0.498	-0.032	0.505	0.026
Hypotension	0.498	-0.045	0.503	-0.185
Antithrombotic agents heparin group	0.498	0.132	0.502	-0.333
Antibiotic resistant infection	0.498	0.090	0.502	-0.291
Wound infection	0.498	-0.239	0.502	-0.278
Viral hepatitis	0.498	-0.038	0.500	-0.090
IV medication	0.498	-0.294	0.500	-0.110
Hip fracture in last 180 days	0.498	0.128	0.497	0.074
Back pain	0.497	0.097	0.502	-0.069
Days psychological therapy	0.497	0.320	0.502	-0.555
Deep vein thrombosis	0.497	-0.080	0.499	-0.171
Fall-related injury (HILMO)	0.497	0.131	0.496	0.012
Cancer	0.496	-0.043	0.508	-0.222
Other fracture in last 180 days	0.496	-0.030	0.503	-0.496
Multi-morbidity cluster	0.496	0.229	0.500	-0.109
Cataracts	0.495	-0.005	0.498	-0.089
Preferred activity setting - inside facility or off unit	0.494	-0.066	0.518	0.113
Memory donepezil	0.494	0.042	0.511	0.169
Repetitive verbalizations of depression, anxiety and sad mood (3 cat)	0.493	0.040	0.521	-0.344
Preferred activity - music	0.493	0.018	0.510	0.065
Days physical therapy	0.493	-0.199	0.505	-0.391
allergies	0.493	0.067	0.504	-0.183
Urinary tract infection in last 30 days	0.493	0.014	0.501	0.181
Preferred activity - helping others	0.493	0.081	0.494	-0.026
Memory memantine	0.492	-0.049	0.535	0.295
Hip fracture	0.492	-0.024	0.510	-0.140
Preferred activity setting - outside facility	0.492	0.006	0.500	0.044
verbally abusive symptoms - change	0.491	0.041	0.528	-0.223
Joint other than hip pain	0.491	-0.004	0.501	0.044
Congestive heart failure	0.491	0.036	0.498	-0.033
Hypnotics and sedatives melatonin	0.490	0.017	0.509	0.036
Delirium - mental function varies over course of day	0.490	-0.002	0.501	0.049
Antidepressants other	0.490	-0.022	0.498	0.061
Dementia other than Alzheimer	0.488	0.002	0.525	-0.190

Hypothyroidism	0.488	0.020	0.494	-0.052
Memory N06D	0.487	0.008	0.543	0.318
Mins physical therapy	0.487	-0.170	0.490	-0.125
Rehabilitation - communication	0.486	0.017	0.511	-0.035
Hallucinations	0.485	0.014	0.536	-0.358
Unpleasant mood in morning (3 cat)	0.485	0.020	0.520	-0.157
Hypertension	0.483	-0.016	0.501	-0.066
CHESS	0.481	-0.091	0.513	0.199
Resident cognitive status, skills or abilities have changed	0.480	-0.090	0.519	0.309
Preferred activity setting - own room	0.478	-0.044	0.515	-0.199

Abbreviations: AUROC: Area Under the Receiver Operating Characteristic. BMI: Body Mass Index, CPS: Cognitive Performance Scale, ADLH: Activity of Daily Life Hierarchy, DRS: Depression Rating Scale, ABS: Aggressive Behavioural Scale, ER: Emergency Room.

Table S2. Confounders.

The confounders of causal modelling selected by a group of three study researchers, experts from the field of clinical geriatrics and pharmachology.

N	Confounder	Operationalization	Explanation
0	Age	continuous	years at baseline assessment
1	Gender	binary	0: male and 1: female
2	BMI	continuous	BMI at baseline assessment
3	Number of drugs	continuous	Number of distinct ATC codes at baseline assessment
4	Number of comorbidities	continuous	Number of distinct disease codes RAI-LTC (MDS) I1A-I2L at baseline assessment
5	CPS	continuous	Scale 0-6: Higher scores indicate more severe cognitive impairment
6	ADLH	continuous	Scale 0-6: Higher scores indicate greater decline (progressive loss) in ADL performance
7	DRS	continuous	Scale 0-14: A score of 3 or more may indicate a potential or actual problem with depression
8	ABS	continuous	Scale 0-12 Higher scores indicate higher levels of aggressive behaviour.
9	Delirium scale	binary	Easily distracted OR periods of altered perception OR awareness of surroundings OR episodes of disordered speech OR periods of restlessness OR periods of lethargy
10	Delusions OR hallucinations	binary	Hallucinations OR delusions
11	Unsteady gait	binary	0: no and 1: yes
12	Flare-up or monitoring of acute condition	binary	Acute episode OR flare-up of recurrent OR chronic problem OR Monitoring acute medical condition
13	Hospital or ER visits (last 6 months)	binary	Hospital stays OR Emergency room visits
14	Chemotherapy or end stage disease	binary	Chemotherapy OR end stage disease
15	Problems with eating and swallowing	binary	0: no and 1: yes
16	Any restrains used	binary	Full bed rails on all sides OR Other types of bed rails OR Trunk restraint OR Limb restraint
17	Physician visits, doctors orders changed or abnormal labs	binary	Physician visits in last 14 days OR Days doctor orders changed in last 14 days OR Abnormal lab values in last 90 days

Abbreviations: BMI: Body Mass Index, CPS: Cognitive Performance Scale, ADLH: Activity of Daily Life Hierarchy, DRS: Depression Rating Scale, ABS: Aggressive Behavioural Scale, ER: Emergency Room.

Table S3. Correlation values of the estimated treatment effects.

Correlation values between the individual treatment effect estimates of double machine learning (DML), double robust (DR) learner, X learner and Causal forest of test data.

	DML	DR-learner	X-learner	Causal forest
DML	1.000	0.915	0.873	0.769
DR-learner	0.915	1.000	0.847	0.707
X-learner	0.873	0.847	1.000	0.788
Causal forest	0.769	0.707	0.788	1.000

Table S4. Sensitivity analysis 1. Average treatment effect (ATE), area under uplift curve (AUUC) and c-for-benefit values for double machine learning (DML), double robust (DR) learner, X learner and causal forest models. Data randomly split for training and testing sets.

Algorithm	Random Treatment, ATE [CI95%]	Random outcome, ATE [CI95%]	Random confounder, ATE [CI95%]
DML	-0.124 [-0.237,-0.015]	0.063 [0.06, 0.065]	0.542 [0.535, 0.55]
DR learner	-0.117 [-0.228,0.003]	0.039 [0.037, 0.042]	0.531 [0.523, 0.539]
X-learner	-0.125 [-0.204,-0.047]	0.062 [0.06, 0.064]	0.551 [0.544, 0.557]
Causal forest	-0.125 [-0.175,-0.076]	0.087 [0.085, 0.09]	0.579 [0.572, 0.587]

Table S5. Sensitivity analysis 2. Average treatment effect (ATE) and 95% confidence intervals (CI) for double machine learning (DML), double robust (DR) learner, X learner, and Causal forest of test data when actual treatment variable or outcome was replaced with a random variable or a random confounder was added in the model.

**Algorithm**	**Random Treatment, ATE [CI95%]**	**Random outcome, ATE [CI95%]**	**Random confounder, ATE [CI95%]**
DML	0.0 [-0.113, 0.113]	0.001 [-0.112, 0.113]	-0.133 [-0.251, -0.019]
DR learner	0.007 [-0.103, 0.147]	0.008 [-0.115, 0.129]	-0.127 [-0.256, -0.009]
X-learner	0.006 [-0.077, 0.089]	0.003 [-0.077, 0.083]	-0.125 [-0.205, -0.045]
Causal forest	0.006 [-0.043, 0.056]	-0.001 [-0.052, 0.051]	-0.116 [-0.166, -0.067]
